# Benevolence – Associations With Stress, Mental Health, and Self-Compassion at the Workplace

**DOI:** 10.3389/fpsyg.2021.568625

**Published:** 2021-06-01

**Authors:** Christina Andersson, Cecilia U. D. Stenfors, Peter Lilliengren, Stefan Einhorn, Walter Osika

**Affiliations:** ^1^Center for Psychiatry Research, Department of Clinical Neuroscience, Karolinska Institute, Stockholm, Sweden; ^2^Center for Social Sustainability, Department of Neurobiology, Care Sciences and Society, Karolinska Institute, Stockholm, Sweden; ^3^Department of Psychology, Stockholm University, Stockholm, Sweden; ^4^Aging Research Center, Department of Neurobiology, Care Sciences and Society, Karolinska Institute, Stockholm, Sweden; ^5^Department of Health Care Sciences, St. Lukas Educational Institute, Ersta Sköndal Bräcke University College, Stockholm, Sweden; ^6^Department of Oncology-Pathology, Karolinska Institute, Stockholm, Sweden; ^7^Stockholm Health Care Services, Center for Psychiatry Research, Department of Clinical Neuroscience, Karolinska Institute, Stockholm, Sweden

**Keywords:** benevolence, stress, self-compassion, well-being, workplace, mental health

## Abstract

**Objective:**

Benevolence is an emerging concept in motivation theory and research as well as in on pro-social behavior, which has stimulated increasing interest in studying factors that impair or facilitate benevolence and effects thereof. This exploratory study examines the associations between benevolence, stress, mental health, self-compassion, and satisfaction with life in two workplace samples.

**Methods:**

In the first study *n* = 522 (38% = female, median age = 42) participants answered questionnaires regarding self-reported stress symptoms (i.e., emotional exhaustion), depressive symptoms and benevolence. In the second study *n* = 49 (female = 96%) participants answered questionnaires regarding perceived stress, self-compassion, anxiety, depression symptoms, and benevolence.

**Results:**

In study 1, measures of emotional exhaustion (*r* = −0.295) and depression (*r* = −0.190) were significantly negatively correlated with benevolence. In study 2, benevolence was significantly negatively correlated with stress (*r* = −0.392) and depression (*r* = −0.310), whereas self-compassion (0.401) was significantly positively correlated with benevolence. While correlations were in expected directions, benevolence was not significantly associated with Satisfaction with Life (*r* = 0.148) or anxiety (*r* = −0.199) in study 2.

**Conclusion:**

Self-assessed benevolence is associated with levels of perceived stress, exhaustion, depression, and self-compassion. Future studies are warranted on how benevolence is related to stress and mental ill health such as depression and anxiety, and if benevolence can be trained in order to decrease stress and mental ill health such as depression and anxiety in workplace settings.

## Introduction

Stress-related problems are global phenomena (OECD, 2013; Vigo et al., 2016) and stress-related health problems (OECD, 2010) and sick-leave (The Swedish Social Insurance Agency, 2017) are also common in Sweden. It entails substantial individual costs in terms of reduced wellbeing and functioning, as well as societal costs in terms of lost productivity, provision of health care and economic support (Ekman et al., 2013; Jonsdottir et al., 2017).

Many employees report high levels of stress in the workplace, which affects the business as well as the working environment (Ishtiak-Ahmed et al., 2014; Savic et al., 2017; The Swedish Social Insurance Agency, 2020). Stress in the workplace is connected to e.g., levels of demand and control (Karasek et al., 1982), support (Turner et al., 2012), effort reward imbalance (Siegrist, 1996), high work pace, constant change, increasing expectations for self-actualization, increasing reliance on interpersonal coordination in the execution of work tasks, and increasing job insecurity (Seidler et al., 2014). Hence, the psychosocial conditions in an organization plays a crucial role on the individuals’ mental health and productivity (Magnusson Hanson et al., 2008; Stenfors et al., 2013).

Stress affects people in different ways, and several definitions of stress have been proposed (Folkman et al., 1986; McEwen, 1998, 2007; Lupien et al., 2009). For example, Lazarus and Folkman (1987) defined stress as a perceived imbalance between demands and resources. Ursin and Eriksen (2010) further argued that the stress response is a healthy and necessary response to something that is perceived as a threat or a challenge, in the cognitive activation theory of stress (CATS). Brosschot et al. (2005) highlighted the detrimental effects regarding prolonged activation of the stress response. They argue that prolonged physiological stress activity can be noticed in (1) Anticipatory responses to potential stressors, (2) Slow recovery from stressors, and (3) Recurrent activity related to past stressors. The stress response can hence be a reaction to something that happens in a specific situation, but it can also be a response to a (sometimes unconscious) ongoing mental process, over a long time period. Brosschot et al. (2018) further argue that it is not the stressor that triggers the stress response, but instead that all humans have a default stress response which is inhibited when safety is perceived generalized unsafety theory of stress (GUTS).

Benevolence is an emerging concept in e.g., motivation theory and research on altruism, pro-social behavior and psychological wellness. One definition of benevolence is the sense of *being able to give* (Martela and Ryan, 2015). Martela and Ryan (2015) developed and studied a new sub-scale comprising four items about benevolence in relation to well-being and mood, in four different sub-studies. It is a complement to the already established three dimensions of internal motivation; autonomy, competence, and relatedness, according to Self Determination Theory (Deci and Ryan, 1985; Ryan and Deci, 2000).

Research is still lacking regarding what factors are of importance for benevolence and how it is related to other aspects of mental health. According to prior research on stress and burn-out, employees are reporting emotional exhaustion, depersonalization, and reduced personal accomplishment when they reach a level of work-related burnout (Maslach and Jackson, 1981; Maslach et al., 2001; Maslach and Leiter, 2017), which hypothetically could decrease benevolence. It has been suggested but is not known if benevolence may act as a buffer against stress-related problems.

The ability to share the feelings of others is often referred to as empathy, and the ability to care for and show concern for others is the core aspect of compassion (Goetz et al., 2010; Singer and Klimecki, 2014). Recent descriptions and research on compassion includes, e.g., evolutionary theory, affective and cognitive neuroscience and attachment psychology, where all humans are thought to have an innate caring motivation to respond to the ones, they are close to, if they are in need (Gilbert, 2010). In a prior study (Andersson et al., 2020 submitted), a self-compassion intervention was shown to have a significant impact on decreasing stress. One definition of self-compassion is how you relate to yourself in a time of suffering (Neff, 2003b) and it is related to a decrease in stress and psychological ill-health, as anxiety and depression (MacBeth and Gumley, 2012; Muris et al., 2016; Kirby et al., 2017; Cuppage et al., 2018). Also, compassion directed toward others have been found to have a buffering effect against stress (Cosley et al., 2010). Experiencing stress or burnout is associated with compassion fatigue (Joinson, 1992) and empathy fatigue (Singer and Klimecki, 2014) and these mental states (that in themselves are related to stress) could have an impact on benevolence.

### Aim of Study

The aim of study 1 was to investigate how benevolence, measured by a newly developed short scale, was associated with stress in terms of emotional exhaustion and depression in a large workplace sample.

The aim of study 2 was to examine if the results from study 1 was replicated in an additional sample, as well as to explore additional associations with self-compassion and satisfaction with life.

### Research Questions and Hypotheses

Is there a relationship between the levels of benevolence, perceived stress, emotional exhaustion, depression, and self-compassion? Based on theory and previous research we expect benevolence to be negatively associated with measures of stress and mental health and positively associated with self-compassion.

## Materials and Methods

### Design

Two cross sectional studies were performed on two independent samples from two different populations.

### Participants

#### Study 1

The sample included office workers in four Swedish organizations. All employees (1,194) were invited to answer an online survey including questions about their work situation, health and wellbeing (MBI-EE and SCL-CD6), out of which 44% responded to the survey, with, *n* = 522, responding to the benevolence questions (BS) (female = 40% age: 24–66, median age = 42).

#### Study 2

This study involved 47 (97% female) participants that were recruited from a public office Social Insurance Agency and a commercial bank to participate in an intervention study evaluating the effects of a compassion training program (Andersson et al., in preparation). When entering the trial, the participants answered questionnaires (using paper and pen) regarding perceived stress (PSS14), self-compassion (SCS), anxiety and depression (HADS), satisfaction with life (SWLS), and benevolence (BS).

### Materials/Instruments

#### Description of the Benevolence Subscale (BS)

Martela and Ryan (2015) developed a brief scale to assess beneficence satisfaction, or the feeling that one has been benevolent. This was to capture what has been called the immediate “warm glow” associated with acts of kindness (Andreoni, 1990). Benevolence is evaluated on a scale ranging from 1 (not at all true) to 7 (very true) and the items are the following (1) *“I feel that my actions have a positive impact on the people around me,”* (2) *“The things I do contribute to the betterment of society,”* (3) *“I have been able to improve the welfare of other people,”* (4) *“In general, my influence in the lives of other people is positive.”*

The translation of the scale from English to Swedish was conducted according to Streiner and Norman (2008). Two different bilingual (Swedish/English) persons with Swedish as a mother tongue translated the scale to Swedish from its original English version (Martela and Ryan, 2015). The translations were then back-translated into English by two other bilingual persons who were not familiar with the original BS measure or its purpose. An analysis group then compared the original and the two back-translated scales, aiming at eliminating any differences between the separate versions. Cronbach’s alpha in study 1 was 0.85 and in study 2 was 0.84.

#### Symptom Checklist, Core Depression Subscale (SCL-CD6)

This scale is a subscale of the SCL-90, which measure core psychological and physical symptoms of depression during the last week. It has been validated against other comprehensive measures of clinical depression and found to have good validity and psychometric properties (Magnusson Hanson et al., 2014). The level of depressive symptoms is rated on a 5-point Likert scale ranging from 1 to 5, reflecting the perceived impact of symptoms in the previous week. Examples of items from the scale includes *“blaming yourself for things”* or *“worrying too much about things.”* Reliability in the current sample of study 1 is a Cronbach’s alpha of 0.88.

#### Maslach Burnout Inventory, Emotional Exhaustion Subscale (MBI-EE)

The scale is a subscale from the Maslach Burnout Inventory general survey (MBI-GS) (Schaufeli et al., 1996; Schutte et al., 2000). The subscale has five items, and two examples are *“My job makes me feel emotionally drained”* or *“I feel burned out by work.”* The score is from 1 (a few times a year or less/never) to 6 (every day). The scale has good internal consistency with a reliability of 0.86 (Cronbach’s alpha) in the current sample of study 1.

#### Perceived Stress Scale (PSS14)

Perceived Stress Scale is a widely used instrument to assess perceived stress. It is developed by Cohen et al. (1983). The original version of 14 items (Cronbach’s α = 0.75). The scale consists of questions regarding thoughts and feelings over the last month, rated on a 5-point Likert scale (0 = *Never*, 4 = *Very often*). An example question is *“In the last month, how often have you felt that you were unable to control the important things in your life?”* The maximum total score is 56, the minimum total score is 0. The scale includes reversed items. The Swedish translation used here has been developed by Eskin and Parr (1996). Reliability in the current sample in study 2 was 0.92.

#### Satisfaction With Life Scale (SWLS)

The satisfaction with life scale is developed by Diener et al. (1985). It’s a 5-item questionnaire to measure subjective well-being in one’s life as a whole. Each item is rated on a 7-point Likert scale (1–7), with 1 = *strongly disagree* and 7 = *strongly agree*. The scale has good psychometric properties with internal consistency reliability (Cronbach’s α = 0.88) Kobau et al. (2010). Reliability in the current sample of study 2 is 0.87.

#### The Hospital Anxiety and Depression Scale (HAD-S)

The Hospital Anxiety and Depression Scale was developed by Zigmond and Snaith (1983) and is a 14-item questionnaire to measure self-reported anxiety and depression scores. Seven items target anxiety (HADS-A) and seven items depression (HADS-D). Each item is rated on a 4-point Likert scale (0–3) with 0 = *never* to 3 = *very often*. The total score on each respective subscale is 21. A score over 15 is interpreted as severe depression or anxiety. A score above 11 is interpreted as the individual likely having a problem related to anxiety or depression and a score around 8–10 is seen as threshold value. Test-retest reliability has been found to be strong, 0.84 for anxiety and 0.71 for depression (Andersson et al., 2003) and internal consistency (Cronbach’s alpha) between 0.68 and 0.93 for the anxiety scale and between 0.67 and 0.90 for the depression scale. Reliability in the current sample of study 2 is 0.93 for HADS total and 0.86 for the anxiety scale and 0.89 for the depression scale.

#### Self-Compassion Scale (SCS)

This 26-items scale assesses levels of self-compassion (Neff, 2003a). There are three factors of positive self-compassion: Self-kindness, Common humanity and Mindfulness, and three factors that focus on a lack of self-compassion: Self-judgment, Isolation, and Over-identification. Participants indicate how often they engage in these ways of self-relating in times of distress rated on a Likert scale 1–5. The scale includes reversed items. The scale has good reliability (Cronbach’s alphas ranging from 0.75 to 0.81). Reliability in the current sample of study 2 is 0.92.

### Ethical Considerations

Both studies were approved by the regional Ethical Review Board in Stockholm, Sweden (Dnr 2014/1300-31/5 and Dnr 2015/1589-31/5).

### Statistical Analyses

The statistical analyses in study 1 and 2 were performed in SPSS 25. Bivariate Pearson *r* correlations were calculated between study measures. Listwise deletion was applied in the analyses. Before analyses, all variables were screened for normality. Due to the exploratory aim of the study (Bender and Lange, 2001), the two-way significance level was set to 0.05 without correction for familywise error rate (e.g., Bonferroni).

## Results

### Study 1

Results are displayed in Table 1. Benevolence was associated with measures of psychological functioning and well-being, in that higher levels of benevolence were related to significantly less emotional exhaustion (*r* = −0.295, *p* < 0.01) and less depressive symptoms (*r* = −0.190, *p* < 0.01). Partial correlations with adjustment for age and gender showed similar results.

**TABLE 1:** Study 1: Descriptive statistics and correlations.

				**Correlations**		
	***n***	***M***	***SD***	**1**	**2**	**3**
1. Benevolence	522	3.75	0.79	–		
2. SCL-CD6	522	1.84	0.77	−0.190**	–	
3. MBI-EE	521	2.15	1.04	−0.295**	0.619**	–

SCL-CD6, symptom checklist, core depression subscale; MBI-EE, Maslach Burnout Inventory, emotional exhaustion subscale. **p < 0.01 (two-tailed).

### Study 2

Results are displayed in Table 2. In this sample, we found that higher levels of benevolence were significantly associated with lower levels of perceived stress (*r* = −0.392, *p* = 0.006) depression (*r* = −0.310, *p* = 0.034) and higher levels of self-compassion (*r* = 0.401, *p* = 0.005). The association with anxiety was negative (*r* = −0.199) but did not reach significance (*p* = 0.180). Similarly, the association with Satisfaction with Life was positive, but not significant (*r* = 0.148 *p* = 0.322).

**TABLE 2:** Study 2: Descriptive statistics and correlations.

				**Correlations**					
	***n***	***M***	***SD***	**1**	**2**	**3**	**4**	**5**	**6**
1. Benevolence	47	15.7	2.6	−					
2. PSS14	47	27.3	10.2	−0.392**	−				
3. SWLS	47	24.3	6.30	0.148	−0.557**	−			
4. HADS-D	47	4.60	4.13	−0.310*	0.689**	−0.690**	−		
5. HADS-A	47	7.45	4.96	–0.199	−712**	−0.575**	0.758**	−	
6. SCS	47	79.8	18.1	0.401**	−0.652**	0.337*	−0.457**	−0.570**	−

PSS14, perceived stress scale; SWLS, satisfaction with life scale; HADS-D, the hospital anxiety and depression scale, depression; HADS-A, the hospital anxiety and depression scale, anxiety; SCS, self-compassion scale. *p < 0.05; **p < 0.01 (two-tailed).

## Discussion

The aim of this study was to investigate the associations between stress, mental health, self-compassion and benevolence in two independent samples recruited from several Swedish workplaces. In study 1, a negative correlation between levels of self-rated stress, depression and benevolence was found. This was corroborated in the second study, where a similar pattern of associations between measures of perceived stress, depression, anxiety and benevolence were seen. Significant correlations were in the “moderately strong” range (e.g., 0.30–0.50) according to Cohen (1992), suggesting meaningful connections between concepts. While the correlations between benevolence, anxiety and satisfaction with life were not significant in Study 2, they were in the expected directions and non-significance may reflect limited power.

In study 2, we also found a positive relationship between benevolence and self-compassion, which also exhibited negative correlations with stress, depression and anxiety. Hence, self-compassion was associated with psychological well-being, which confirms earlier findings (Neff, 2003b; Kirby et al., 2017). Benevolence does not target suffering explicitly as self-compassion but being benevolent has been suggested to be an innate quality in all humans (Keltner et al., 2010). Self-compassion has been described to target, e.g., self-criticism and subsequent suffering (Gilbert and Procter, 2006), and it might be easier to access a state of benevolence if one has self-compassion, not being so hard on oneself and being able to see the positive impact one has on others. The results also suggest that benevolence could have an impact on the mental health and wellbeing outcomes. One could argue that there may be a reciprocal/bidirectional relationship. For example, stress and depression could reduce benevolence, but high benevolence could also have a reducing and/or buffering effect on stress and depression (Piliavin and Siegl, 2007). Acting benevolent involves both the giving end and the receiving end of the flow of pro-social and compassion behavior. Therefore, it would be of interest to study how it relates to the fears in each direction of the compassionate flow and see if decreasing the fears and blocks (Kirby et al., 2019) will have a positive impact on benevolence.

The results in study 2 were in line with the results in study 1. In both studies the Benevolence scale was used, while different measures of stress and depression were used in the two studies due to the different study settings (i.e., workplace setting in study 1 and a psychological intervention trial in study 2 where HAD-S is more frequently used in clinical settings). The correlations between benevolence and depression (in study 1 measured by SCL-CD6 and in study 2 measured by HAD-S) were however similar. Furthermore, prior works have found the two depression measures to correlate highly (Bjelland et al., 2002). The results from study 1 and 2 together thus provide robust support to benevolence being negatively associated with depressive symptoms. Different and complimentary aspects of stress were investigated in study 1 and 2. Symptoms of chronic stress were investigated in study 1 in terms of emotional exhaustion symptoms (MBI-EE), while perceived stress (PSS14) was investigated in study 2 which tap the experience of stressors in terms of stressful situations and ability to cope with those stressors. The results from study 1 and 2 are thus complimentary and suggest that a higher degree of benevolence is associated with a lower level of both perceived stressors and ability to cope (PSS14) as well as symptoms of chronic stress in the form of emotional exhaustion symptoms.

Neff and Vonk (2009) highlight the difference between self-compassion and self-esteem, the latter being more unstable and often including the comparison of oneself against others, which can affect a person’s self-worth. Hallsten et al. (2005) also connects the same aspect of a performance-based self-esteem relationship to emotional exhaustion. Even if acute stress has been described to trigger pro-social behavior (Raposa et al., 2016) chronic stress on the other hand have been shown in several studies to increase the risk for both mental (Grossi et al., 2015) and e.g., cardiovascular diseases (Steptoe and Kivimäki, 2012).

According to the Self Determination Theory (SDT), individuals are motivated by extrinsic and intrinsic factors. Extrinsic motivation refers to behaviors driven by external rewards like money, praise or fame. Intrinsic motivation refers to behaviors driven by growth and internal satisfaction (Deci and Ryan, 1985; Kasser and Ryan, 1996). The SDT recognizes three basic psychological needs which drives our behavior, autonomy, competence and relatedness. Martela and Ryan (2015, 2020) investigated if benevolence could be categorized as an additional basic psychological need, but concluded that it didn’t qualify as such, but rather as a psychological factor which e.g., could enhance wellbeing. Another aspect of benevolence and its association to stress and motivation is Kaufman (2018) description of the concept of self-actualization, which involves helping others and not only seeing self-actualization as an individual project. The role of benevolence as a mental state, and if it relates to e.g., actual pro-social behavior still needs to be clarified.

### Limitations

Some limitations do apply to the current study: First, both studies were cross-sectional and hence correlational, and thus, no causal inferences can be made. Secondly, both samples were limited to certain populations—office workers in four different organizations in study 1, and self-selected participants from different workplaces to an intervention program in study 2. Future studies that use experimental designs would be needed to any establish causal links between these factors, e.g., by investigating the effects of increased benevolence on stress and other mental health outcomes in interventions studies. Third, all the participants were from Sweden and were recruited either from a survey targeted at specific workplaces (study 1) or from intervention studies targeted at specific working populations (study 2). In order to validate the results from the current study, more studies including samples from e.g., other countries and cultural backgrounds are needed. Fourth, age was not registered in study 2, which limits the generalizability. All the participants were in the age range 25–65 years. Finally, the outcome measures were limited to self-assessment scales, and in order to further investigate the validity of the associations, it would be valuable to add measures of behavioral and physiological variables.

## Conclusion

This is the first study to our knowledge to show that there is an association between self-reported stress levels and benevolence, as well as between self-compassion and benevolence. Future intervention studies should investigate if it is possible to change levels of benevolence and its effect on outcomes such as collaboration, innovation and performance (Condon, 2019). Future studies could also look at the concept of organizational virtuousness and benevolence and its effect on creating a positive organizational culture but also how it affects turnover rate and well-being (Cameron and Caza, 2004). In future studies it would also be of interest to include the “reduced personal accomplishment” aspect of burnout from the MBI scale, to investigate its relationship with the benevolence scale.

## Data Availability Statement

The raw data supporting the conclusions of this article will be made available by the authors, without undue reservation.

## Ethics Statement

The studies involving human participants were reviewed and approved by the regional Ethical Review Board in Stockholm, Sweden (Dnr 2014/1300-31/5 and Dnr 2015/1589-31/5). The patients/participants provided their written informed consent to participate in this study.

## Author Contributions

CA, WO, and SE designed study 1. CA sampled the data. PL analyzed the data, in collaboration with CA. CS and WO designed study 2 and selected the study measures. CS sampled the data and analyzed the data in study 2. All authors contributed to the article and approved the submitted version.

## Conflict of Interest

The authors declare that the research was conducted in the absence of any commercial or financial relationships that could be construed as a potential conflict of interest.

## References

[B1] Andersson C. Bergsten-Lindert K. Lilliengren P. Norback K. Rask K. Einhorn S. (2020). The effectiveness of smartphone compassion training on stress among university students: a pilot randomized trial. *J. Clin. Psychol.* 77 927–945 10.1002/jclp.23092 33245161

[B2] Andersson G. Kaldo-Sandström V. Strömma L. Strömgren T. (2003). Internet administration of the hospital anxiety and depression scale in a sample of tinnitus patients. *J. Psychosom. Res.* 55 259–262. 10.1016/S0022-3999(02)00575-5 12932800

[B3] Andreoni J. (1990). Impure altruism and donations to public goods: a theory of warm glow giving. *Econ. J.* 100 464–477. 10.2307/2234133

[B4] Bender R. Lange S. (2001). Adjusting for multiple testing - when and how? *J. Clin. Epidemiol.* 54 343–349. 10.1016/S0895-4356(00)00314-0 11297884

[B5] Bjelland I. Dahl A. A. Haug T. T. Neckelmann D. (2002). The validity of the hospital anxiety and depression scale: an updated literature review. *J. Psychosom. Res.* 52 69–77. 10.1016/S0022-3999(01)00296-3 11832252

[B6] Brosschot J. F. Pieper S. Thayer J. F. (2005). Expanding stress theory: prolonged activation and perseverative cognition. *Psychoneuroendocrinology* 30 1043–1049. 10.1016/j.psyneuen.2005.04.008 15939546

[B7] Brosschot J. F. Verkuil B. Thayer J. (2018). The Generalized Unsafety Theory of Stress (GUTS) and why the stress response is fundamentally unconscious. *Psychosom. Med.* 80 A155–A155.

[B8] Cameron K. S. Caza A. (2004). Introduction: contributions to the discipline of positive organizational scholarship. *Am. Behav. Sci.* 47 731–739. 10.1177/0002764203260207

[B9] Cohen J. (1992). A power primer. *Psychol. Bull.* 112 155–159. 10.1037/0033-2909.112.1.155 19565683

[B10] Cohen S. Kamarck T. Mermelstein R. (1983). A global measure of perceived stress. *J. Health Soc. Behav.* 24:385. 10.2307/2136404 6668417

[B11] Condon P. (2019). Meditation in context: factors that facilitate prosocial behavior. *Curr. Opin. Psychol.* 28 15–19. 10.1016/j.copsyc.2018.09.011 30359936

[B12] Cosley B. J. McCoy S. K. Saslow L. R. Epel E. S. (2010). Is compassion for others stress buffering? Consequences of compassion and social support for physiological reactivity to stress. *J. Exp. Soc. Psychol.* 46 816–823. 10.1016/j.jesp.2010.04.008

[B13] Cuppage J. Baird K. Gibson J. Booth R. Hevey D. (2018). Compassion focused therapy: exploring the effectiveness with a transdiagnostic group and potential processes of change. *Br. J. Clin. Psychol.* 57 240–254. 10.1111/bjc.12162 29044607

[B14] Deci E. L. Ryan R. M. (1985). *Intrinsic Motivation and Self-Determination in Human Behavior.* New York, NY: Plenum. 10.1007/978-1-4899-2271-7

[B15] Diener E. Emmons A. R. Larsen J. R. Griffin S. (1985). The satisfaction with life scale. *J. Personal. Asses.* 71–75. 10.1207/s15327752jpa4901_13 16367493

[B16] Ekman M. Granström O. Omérov S. Jacob J. Landén M. (2013). The societal cost of depression: evidence from 10 000 Swedish patients in psychiatric care. *J. Affect. Disord.* 150 790–797. 10.1016/j.jad.2013.03.003 23611536

[B17] Eskin M. Parr D. (1996). *Introducing a Swedish Version of an Instrument Measuring Mental Stress*. Stockholm: University Department of Psychology.

[B18] Folkman S. Lazarus R. S. Gruen R. J. DeLongis A. (1986). Appraisal, coping, health status, and psychological symptoms. *J. Pers. Soc. Psychol.* 50 571–579. 10.1037/0022-3514.50.3.571 3701593

[B19] Gilbert P. (2010). *The Compassionate Mind: A New Approach to Life’s Challenges.* London: Constable & Robinson.

[B20] Gilbert P. Procter S. (2006). Compassionate mind training for people with high shame and self−criticism: overview and pilot study of a group therapy approach. *Clin. Psychol. Psychother.* 13 353–379. 10.1002/cpp.507

[B21] Goetz J. L. Keltner D. Simon-Thomas E. (2010). Compassion: an evolutionary analysis and empirical review. *Psychol. Bull.* 136 351–374. 10.1037/a0018807 20438142 PMC2864937

[B22] Grossi G. Perski A. Osika W. Savic I. (2015). Stress−related exhaustion disorder–clinical manifestation of burnout? A review of assessment methods, sleep impairments, cognitive disturbances, and neuro−biological and physiological changes in clinical burnout. *Scand. J. Psychol.* 56 626–636. 10.1111/sjop.12251 26496458

[B23] Hallsten L. Josephson M. Torgén M. (2005). Performancebased self-esteem: a driving force in burnout processes and its assessment. *Work and Health.* 4 1–40.

[B24] Ishtiak-Ahmed K. Perski A. Mittendorfer-Rutz E. (2014). Risk markers of all-cause and diagnosis-specific disability pension – a prospective cohort study of individuals sickness absent due to stress-related mental disorders. *BMC Public Health* 14:805. 10.1186/1471-2458-14-805 25099303 PMC4147178

[B25] Joinson C. (1992). Coping with compassion fatigue. *Nursing* 22 116–120. 10.1097/00152193-199204000-00035 1570090

[B26] Jonsdottir I. H. Nordlund A. Ellbin S. Ljung T. Glise K. Währborg P. (2017). Working memory and attention are still impaired after three years in patients with stress−related exhaustion. *Scand. J. Psychol.* 58 504–509. 10.1111/sjop.12394 29023756

[B27] Karasek R. A. Theorell T. G. Schwartz J. Pieper C. Alfredsson L. (1982). Job, psychological factors and coronary heart disease. Swedish prospective findings and US prevalence findings using a new occupational inference method. *Adv. Cardiol.* 29 62−67. 10.1159/000406198 6977263

[B28] Kasser T. Ryan R. M. (1996). Further examining the American dream: Differential correlates of intrinsic and extrinsic goals. *Pers. Soc. Psychol. Bull.* 22 280–287. 10.1177/0146167296223006

[B29] Kaufman S. B. (2018). Self-actualizing people in the 21st century: integration with contemporary theory and research on personality and well-being. *J. Human. Psychol.* 1–33. 10.1177/0022167818809187

[B30] Keltner D. Marsh J. Adam Smith J. (2010). *Compassionate Instinct. The science of Human Goodness.* New York, NY: Ww Norton Co.

[B31] Kirby J. N. Tellegen C. L. Steindl S. R. (2017). A meta-analysis of compassion-based interventions: current state of knowledge and future directions. *Behav. Ther.* 48 778–792. 10.1016/j.beth.2017.06.003 29029675

[B32] Kirby N. J. Day J. Sager V. (2019). The ‘Flow’ of compassion: a meta-analysis of the fears of compassion scales and psychological functioning. *Clini. Psychol. Rev.* 70:39. 10.1016/j.cpr.2019.03.001 30884253

[B33] Kobau R. Sniezek J. Zack M. M. Lucas R. E. Burns A. (2010). Well−being assessment: an evaluation of well−being scales for public health and population estimates of well−being among US adults. *Appl. Psychol. Health Well being* 2 272–297. 10.1111/j.1758-0854.2010.01035.x

[B34] Lazarus R. S. Folkman S. (1987). Transactional theory and research on emotions and coping. *Eur. J. Pers.* 1 141–169. 10.1002/per.2410010304

[B35] Lupien S. J. McEwen B. S. Gunnar M. R. Heim C. (2009). Effects of stress throughout the lifespan on the brain, behaviour and cognition. *Nat. Rev. Neurosci.* 10 434–445. 10.1038/nrn2639 19401723

[B36] MacBeth A. Gumley A. (2012). Exploring compassion: a meta-analysis of the association between self-compassion and psychopathology. *Clin. Psychol. Rev.* 32 545–552. 10.1016/j.cpr.2012.06.003 22796446

[B37] Magnusson Hanson L. L. Theorell T. Oxenstierna G. Hyde M. Westerlund H. (2008). Demand, control and social climate as predictors of emotional exhaustion symptoms in working Swedish men and women. *Scand. J. Soc. Med.* 36 737–743. 10.1177/1403494808090164 18684778

[B38] Magnusson Hanson L. L. Westerlund H. Leineweber C. Rugulies R. Osika W. Theorell T. (2014). The Symptom Checklist-core depression (SCL-CD6) scale: psychometric properties of a brief six item scale for the assessment of depression. *Scand. J. Public Health* 42 82–88. 10.1177/1403494813500591 23982463

[B39] Martela F. Ryan R. M. (2015). The benefits of benevolence: basic psychological needs, beneficence, and the enhancement of well−being. *J. Pers.* 84 750–764. 10.1111/jopy.12215 26249135

[B40] Martela F. Ryan R. M. (2020). Distinguishing between basic psychological needs and basic wellness enhancers: the case of beneficence as a candidate psychological need. *Motiv. Emot.* 44 116–133. 10.1007/s11031-019-09800-x

[B41] Maslach C. Jackson S. E. (1981). The measurement of experienced burnout. *J. Occup. Behav.* 2 99–113. 10.1002/job.4030020205

[B42] Maslach C. Leiter M. P. (2017). New insights into burnout and health care: strategies for improving civility and alleviating burnout. *Med. Teach.* 39 160–163. 10.1080/0142159X.2016.1248918 27841065

[B43] Maslach C. Schaufeli W. B. Leiter M. P. (2001). Job burnout. *Annu. Rev. Psychol.* 52 397–422. 10.1146/annurev.psych.52.1.397 11148311

[B44] McEwen B. S. (1998). Protective and damaging effects of stress mediators. *N. Engl. J. Med.* 338 171–179. 10.1056/NEJM199801153380307 9428819

[B45] McEwen B. S. (2007). Physiology and neurobiology of stress and adaptation: central role of the brain. *Physiol. Rev.* 87 873–904. 10.1152/physrev.00041.2006 17615391

[B46] Muris P. Meesters C. Pierik A. de Kock B. (2016). Good for the self: Self-compassion and other self-related constructs in relation to symptoms of anxiety and depression in non-clinical youths. *J. Child Fam. Stud.* 25 607–617. 10.1007/s10826-015-0235-2 26834447 PMC4720693

[B47] Neff K. (2003b). Self-compassion: an alternative conceptualization of a healthy attitude toward oneself. *Self Identity* 2 85–102. 10.1080/15298860309032

[B48] Neff K. D. (2003a). The development and validation of a scale to measure self-compassion. *Self and Identity* 2 223–250. 10.1080/15298860309027 26979311

[B49] Neff K. D. Vonk R. (2009). Self-compassion versus global self-esteem: two different ways of relating to oneself. *J. Pers.* 77 23–50. 10.1111/j.1467-6494.2008.00537.x 19076996

[B50] OECD (2010). *Sickness, Disability and Work: Breaking the Barriers. A Synthesis of Findings Across OECD Countries.* Paris: OECD Publishing.

[B51] OECD (2013). *Mental Health and Work.* Sweden: OECD.

[B52] Piliavin J. A. Siegl E. (2007). Health benefits of volunteering in the wisconsin longitudinal study. *J. Health Soc. Behav.* 48 450–464. 10.1177/002214650704800408 18198690

[B53] Raposa B. E. Laws B. H. Ansell B. E. (2016). Prosocial behavior mitigates the negative effects of stress in everyday life. *Clin. Psychol. Sci.* 4 691–698. 10.1177/2167702615611073 27500075 PMC4974016

[B54] Ryan R. M. Deci E. L. (2000). Self-determination theory and the facilitation of intrinsic motivation, social development, and wellbeing. *Am. Psychol.* 55 68–78. 10.1037/0003-066X.55.1.68 11392867

[B55] Savic I. Perski A. Osika W. (2017). MRI shows that exhaustion syndrome due to chronic occupational stress is associated with partially reversible cerebral changes. *Cereb. Cortex* 28 1–13. 10.1093/cercor/bhw413 28108490

[B56] Schaufeli W. B. Leiter M. P. Maslach C. Jackson S. E. (1996). “MBI–general survey,” in *Maslach Burnout Inventory Manual*, 3rd Edn, eds Maslach C. Jackson S. E. Leiter M. P. (Palo Alto, CA: Consulting Pyschologists Press).

[B57] Schutte N. Toppinen S. Kalimo R. Schaufeli W. (2000). The factorial validity of the Maslach Burnout Inventory−General Survey (MBI−GS) across occupational groups and nations. *J. Occup. Organ. Psychol.* 73 53–66. 10.1348/096317900166877

[B58] Seidler A. Thinschmidt M. Deckert S. Then F. Hegewald J. Nieuwenhuijsen K. (2014). The role of psychosocial working conditions on burnout and its core component emotional exhaustion – a systematic review. *J. Occupat. Med. Toxicol.* 9 10. 10.1186/1745-6673-9-10 24628839 PMC4233644

[B59] Siegrist J. (1996). Adverse health effects of high-effort/low-reward conditions. *J. Occup. Health Psychol.* 1 27−41. 10.1037//1076-8998.1.1.27 9547031

[B60] Singer T. Klimecki O. (2014). Empathy and compassion. *Curr. Biol.* 24 R875–R878. 10.1016/j.cub.2014.06.054 25247366

[B61] Stenfors C. U. Hanson L. M. Oxenstierna G. Theorell T. Nilsson L. G. (2013). Psychosocial working conditions and cognitive complaints among Swedish employees. *P*L*oS one* 8:e60637. 10.1371/journal.pone.0060637 23560101 PMC3613346

[B62] Steptoe A. Kivimäki M. (2012). Stress and cardiovascular disease. *Nat. Rev. Cardiol.* 9 360–370. 10.1038/nrcardio.2012.45 22473079

[B63] Streiner D. L. Norman G. R. (2008). *Health Measurement Scales: A Practical Guide to Their Development and Use*, 4th Edn. New York, NY: Oxford University Press. 10.1093/acprof:oso/9780199231881.001.0001

[B64] The Swedish Social Insurance Agency (2017). Psykiatriska diagnoser. Lång väg tillbaka till arbete vid sjukskrivning (psychiatric diagnoses. A long way back to work from sick-leave). *Korta Anal.* 2017:1.

[B65] The Swedish Social Insurance Agency (2020). “Follow-up of the development of sick leave (Uppf ljning av sjukfr nvarons utveckling)”, Report 001382-2020.

[B66] Turner N. Stride C. B. Carter A. J. McCaughey D. Carroll A. E. (2012). Job demands-control-support model and employee safety performance. *Accid Anal. Prev.* 45 811−817. 10.1016/j.aap.2011.07.005 22269573

[B67] Ursin H. Eriksen H. R. (2010). Cognitive activation theory of stress (CATS). *Neurosci. Biobehav. Rev.* 34 877–881. 10.1016/j.neubiorev.2009.03.001 20359586

[B68] Vigo D. Thornicroft G. Atun R. (2016). Estimating the true global burden of mental illness. *Lancet Psychiatry* 3 171−178. 10.1016/S2215-0366(15)00505-2 26851330

[B69] Zigmond A. S. Snaith R. P. (1983). The hospital anxiety and depression scale. *Acta Psychiatr. Scand.* 67 361–370. 10.1111/j.1600-0447.1983.tb09716.x 6880820

